# Planar, Polysilazane‐Derived Porous Ceramic Supports for Membrane and Catalysis Applications

**DOI:** 10.1111/jace.13758

**Published:** 2015-07-20

**Authors:** Thomas Konegger, Lee F. Williams, Rajendra K. Bordia

**Affiliations:** ^1^ Department of Materials Science and EngineeringClemson UniversityClemsonSouth Carolina29634; ^2^ Institute of Chemical Technologies and AnalyticsVienna University of TechnologyVienna1060Austria

## Abstract

Porous, silicon carbonitride‐based ceramic support structures for potential membrane and catalysis applications were generated from a preceramic polysilazane precursor in combination with spherical, ultrahigh‐molecular weight polyethylene microparticles through a sacrificial filler approach. A screening evaluation was used for the determination of the impact of both porogen content and porogen size on pore structure, strength, and permeability characteristics of planar specimens. By optimizing both the composition as well as cross‐linking parameters, maximum characteristic biaxial flexural strengths of 65 MPa and porosities of 42% were achieved. The evolution of an interconnected, open‐pore network during thermal porogen removal and conversion of the preceramic polymer led to air permeabilities in the order of 10^−14^ m². The materials were further exposed to long‐term heat treatments to demonstrate the stability of properties after 100 h at 800°C in oxidizing, inert, and reducing environments. The determined performance, in combination with the versatile preparation method, illustrates the feasibility of this processing approach for the generation of porous ceramic support structures for applications at elevated temperatures in a variety of fields, including membrane and catalysis science.

## 1 Introduction

Porous ceramics have a wide range of applications due to their exceptional properties, including their mechanical strength and their stability in corrosive and high‐temperature environments. Recently, the application of open‐cell ceramic structures in energy‐related applications such as membrane or catalysis science has gained increasing attention. In both fields, porous ceramics have been widely used as supports to either provide a mechanically stable substrate for subsequently deposited thin membrane layers,^1^, ^2^ or a high‐surface area scaffold for catalytically active compounds.^3^, ^4^

Depending on the anticipated application, the generated structures have to fulfill a number of requirements in terms of total porosity, pore structure, surface area, permeability, and mechanical stability. For porous ceramic membrane supports, structures with pore sizes between 1 and 15 μm and interconnected, open porosities between 30% and 45% are desired.^1^, ^5^ The support structure is generally prepared by conventional ceramic processing techniques such as pressing or casting with subsequent consolidation through sintering at high temperatures. In the majority of reports in the literature, Al_2_O_3_ is used as a support material.^6^ A disadvantage of this type of material in combination with selective layers made of other materials such as SiC arises from unwanted interactions between the materials as well as discrepancies in the coefficients of thermal expansion, leading to stresses during thermal cycling. This has resulted in increased interest in developing alternative support materials, including non‐oxide materials such as SiC or Si_3_N_4_.^7^, ^8^, ^9^, ^10^, ^11^, ^12^ However, due to their highly demanding sintering characteristics—more specifically, the need for high processing temperatures as well as required addition of sintering aids—the use of non‐oxide materials for porous support structures remains a technical challenge.

Polymer‐derived ceramics (PDCs) present an alternative processing route for the production of ceramic materials. The use of Si‐based polymers such as polysilazanes or polycarbosilanes as precursors for non‐oxide PDCs obtained through a controlled thermal decomposition process has gained increasing scientific attention in the past decades.^13^, ^14^, ^15^

In addition to significantly lowered consolidation temperatures as well as the ability to produce high‐purity products, a major advantage of this route is the wide variety of shaping options, including the ability to produce coatings,^16^, ^17^ fibers,^18^ tapes,^19^ and tubes.^20^ Due to the polymer nature of precursors, a number of processing methods are available which are not accessible by resorting to conventional powder‐based ceramics. This, in turn, can be adopted for the preparation of porous materials.

A wide variety of techniques exist for the generation of porosity in ceramic materials, which have been the focus of several extensive review articles^21^, ^22^, ^23^ as well as a comprehensive book^3^ in the past. Aside from partial sintering, the most common techniques to produce porous ceramics include the replica method (using polymeric sponges as templates), direct foaming (by introducing of gaseous species), or the use of sacrificial porogens. To a large extent, these techniques have been shown to be applicable to PDCs as well.^24^, ^25^, ^26^

The application of the sacrificial porogen technique in combination with PDCs has been shown to be very versatile as a result of the tailorability of both open‐ and closed‐cell structures, and the high reproducibility of the pore structures generated. In this technique, the preceramic polymer is mixed with sacrificial templates which are extracted during a later step, either by burn‐off, thermal decomposition, etching, or leaching. A wide variety of sacrificial porogens have been used in combination with preceramic polymers, including salts,^27^ silica beads,^28^ wood,^29^ as well as a number of conventional polymers such as PS,^30^, ^31^, ^32^, ^33^, ^34^ PMMA,^35^, ^36^, ^37^ low‐density PE,^38^ or PVA.^39^ Schmalz and co‐authors successfully produced porous polysilazane‐derived silicon carbonitride (SiCN) by using ultra‐high molecular weight polyethylene (UHMW‐PE) as a sacrificial template.^40^ UHMW‐PE was shown to be a very well suited template due its chemical compatibility with polysilazane as a result of its oxygen‐free chemical composition, its full conversion into gaseous species upon thermal decomposition, and its compatibility with the polysilazane precursor during mixing.

In this manuscript, we report on the fabrication of planar, macroporous ceramic support structures by combining a polysilazane precursor with UHMW‐PE microbeads as sacrificial templates. Polysilazane‐derived SiCN is chosen due to its excellent mechanical properties and its oxidation and creep resistance.^41^ In contrast to the majority of previous works employing this combination of materials, the preceramic polymer is used in its initial, liquid state, thus allowing a more versatile shaping through a casting process with subsequent curing. We reported on this processing approach recently, and showed its general feasibility for the preparation of porous, free‐standing structures in the Si–C and Si–C–N systems.^42^ A core objective of this present work is an in‐depth correlation between a number of processing parameters and the resulting materials' performance in terms of permeability and strength, as well as the evaluation of its long‐term stability in oxidizing, neutral, and reducing atmospheres at high temperatures, thus leading to the development of a material system well suited for potential membrane or catalysis applications.

## 2 Experimental Procedure

### 2.1 Materials

A commercially available poly(vinyl)silazane (PSZ; HTT 1800, AZ Electronic Materials, Branchburg, NJ) was used as preceramic polymer. This clear, colorless liquid compound, exhibiting a viscosity of 22 mPa·s at 25°C,^43^ can be described by the general formula [–(R–Si–CH_3_)–(NH)–]_*n*_, with *n *=* *1–20, and *R* representing either H (80 mol%) or a cross‐linking active vinyl group (20 mol%). Cross‐linking of the polymer typically starts at temperatures between 180°C and 200°C, whereas the polymer‐to‐ceramic transformation starts between 400°C and 500°C. To promote cross‐linking at lower temperatures, 1 wt% of dicumyl peroxide (99 %; Acros Organics, Geel, Belgium) was added as an initiator.

Two ultrahigh molecular weight polyethylene (UHMW‐PE) powders with particle sizes of 10 or 30 μm, and molecular weights of 1.8 × 10^6^ or 2.0 × 10^6^ g/mol, respectively, were used as sacrificial fillers (Mipelon PM‐200 or Mipelon XM‐220, both Mitsui Chemicals America, Rye Brook, NY). The filler powders were dried under vacuum at 50°C for 12 h before use.

In addition to variation in the filler particle size (10 or 30 μm, respectively), the sacrificial filler fraction was varied: 20, 30, and 40 vol%. Due to the high sensitivity of the preceramic polymer to air and moisture, all steps prior to thermal treatment were carried out in a high‐purity N_2_ atmosphere glove box and thermal treatments under high‐purity flowing N_2_ atmosphere.

### 2.2 Preparation of Planar Supports

Our processing approach for the preparation of planar polymer‐derived ceramic supports was first described in a recent contribution.^42^ After degassing of the preceramic polymer under vacuum, a corresponding amount of sacrificial filler powders was added, yielding typical mixtures between 12 and 30 g. The mixture was magnetically stirred under vacuum for a minimum duration of 30 min. For each specimen, around 2 mL of the homogenized mixture were cast into a cylindrical, elastomeric mold made of polydimethylsiloxane (MoldMax XLS II, Smooth‐On, Inc., Macungie, PA) with an inner diameter of 18 mm. The samples were cross‐linked at 90°C in flowing N_2_, using a heating rate of 1 K/min, and held at the peak temperature for 16 h. To explore the effect of cross‐linking temperature, samples were also cross‐linked at 105°C or 120°C. The samples were demolded and another cross‐linking step using the same parameters was conducted to ensure homogeneous curing. After cross‐linking, the top and bottom parts of the samples were cut off and the planar surfaces and sides were ground to a 2000 grit finish using silicon carbide paper, yielding plane‐parallel disks with typical thicknesses of 3 to 4 mm.

The pyrolytic conversion was carried out in an alumina tube furnace (Lindberg Blue M HTF5534C; Thermo Scientific, Waltham, MA) in high‐purity flowing N_2_ at 800°C for 4 h. Based on thermogravimetric analysis results [see Section 3.1], an adapted heating regime was set up to account for decomposition and molecular rearrangement processes, thus reducing the tendency of crack formation. A heating rate of 1 K/min was used up to 300°C, with an intermittent hold at 130°C for 2 h. Between 300°C and 800°C, the heating rate was reduced to 0.5 K/min. After completion of the peak temperature step, the samples were cooled with a rate of 1 K/min.

To evaluate the long‐term stability at high temperatures, selected pyrolyzed samples were further heat‐treated in oxidizing (air), inert (flowing high‐purity N_2_), or reducing atmosphere (flowing 95% N_2_/5% H_2_) at 800°C for a duration of 100 h.

### 2.3 Characterization

Thermogravimetric investigations of starting materials and cross‐linked green bodies were carried out in flowing N_2_ atmosphere using a simultaneous thermal analyzer, using a heating ramp of 10 K/min up to 1000°C (STA 409; Netzsch, Selb, Germany). The softening and melting behavior of the sacrificial filler particles was determined by thermo‐mechanical analysis of compacted pellets of the UHMW‐PE particles, using a penetration probe at a load of 1 N (160 kPa) and a heating rate of 2 K/min (Q400; TA Instruments, New Castle, DE).

The cross‐sections of the final samples were investigated using scanning electron microscopy (SEM; S4800, Hitachi, Tokyo, Japan). Mercury intrusion porosimetry (PoreMaster 33; Quantachrome, Boynton Beach, FL) was used to clarify the pore structure and size distribution of pore openings, as well as the skeletal density of the material, which was subsequently used to calculate the total porosity of specimens based on the bulk density obtained from the geometric dimensions and specimen weight after pyrolytic conversion. X‐ray diffraction analysis was carried out with crushed and powdered specimens after pyrolytic conversion and after long‐term postpyrolysis heat‐treatment on a Rigaku Ultima IV diffractometer (Rigaku, The Woodlands, TX) using Cu*K*_α_ radiation.

Permeability and strength testing was conducted on pyrolyzed, disk‐shaped specimens with diameters of 13.6 ± 0.6 mm and heights of 2.3 ± 0.7 mm.

The permeability characteristics of the supports were determined using a capillary flow porometer (CFP‐1100‐AEXS; Porous Materials, Inc., Ithaca, NY) with air at pressure differentials up to 0.1 MPa, employing Darcy's law for the calculation of the permeabilities. The investigated sample area was 78.5 mm^2^. At least two specimens per sample composition were tested for initial screening tests, while 7–10 specimens were used for in‐depth evaluation of promising compositions.

The mechanical strength of the supports was determined by a biaxial flexural strength test using a ball‐on‐three‐balls (B3B) setup geometry. The tests were conducted on a universal testing machine (Model 5582; Instron, Norwood, MA) with steel balls (diameter of 9.525 mm) at a crosshead speed of 0.5 mm min^−1^. The biaxial flexural strength σ_B3B_ was determined and calculated as proposed by Börger et al.,^44^ using Eq. (1).

(1) $$\sigma_{B3B}=f\cdot \frac{F}{t^{2}}$$

where *F* is the maximum applied force before fracture, *t* is the support thickness, and *f* is a dimensionless factor which depends on the loading geometry, the specimen geometry, and the material's Poisson's ratio. The dimensionless factor *f* was calculated for each specimen using an analytical solution given by Börger et al.,^44^ assuming a Poisson's ratio of 0.2 for the materials investigated.

For screening purposes, between two and four specimens were evaluated per sample composition. For the most promising sample composition (30 vol% of 10 μm‐sized fillers), cross‐linked at three distinct temperatures, an in‐depth statistical analysis of strength data was conducted by determination of Weibull parameters using the maximum‐likelihood method as well as calculation of sample size bias and confidence intervals according to EN 843‐5,^45^ testing 12–18 specimens each. The same method was used to evaluate specimens after long‐term postpyrolysis heat treatment.

## 3 Results and Discussion

### 3.1 Cross‐Linking and Pyrolytic Conversion

Rigid, bubble‐free specimens were obtained after cross‐linking of the cast PSZ/UHMW‐PE mixtures. Thorough degassing of the preceramic polymer before use as well as mixing under vacuum was shown to be essential for the suppression of pore formation during cross‐linking. Due to the small difference in densities of the preceramic polymer component (1.02 g/cm^3^) and the UHMW‐PE filler particles (0.94 g/cm^3^) and the high viscosity of the mixtures, no sedimentation was observed during the cross‐linking step, yielding a homogeneous distribution of the filler particles in the PSZ matrix. The initial cross‐linking temperature, 90°C, was chosen to be well below the melting temperature of the UHMW‐PE filler (*T*_m_ = 138°C).

**Figure 1:**
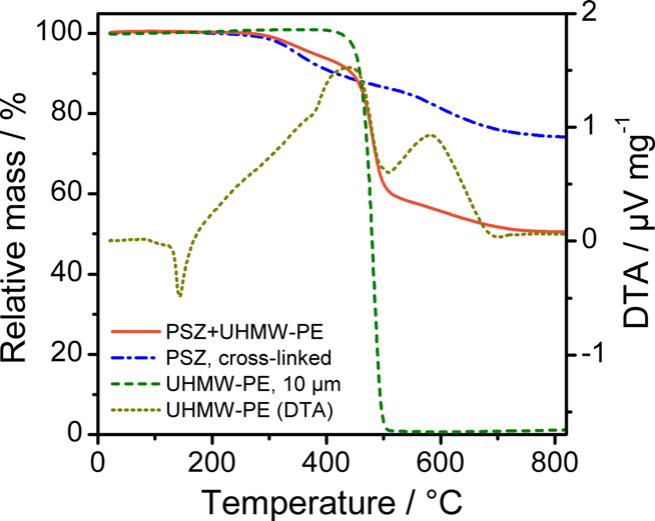
Simultaneous thermal analysis (TG/DTA) of a cross‐linked PSZ/UHMW‐PE specimen (30 vol% UHMW‐PE, 10 μm particle size) together with TG data of pure cross‐linked PSZ and UHMW‐PE in flowing N_2_.

After cross‐linking, heat treatment was used for both UHMW‐PE removal as well as for thermal conversion of the preceramic polymer into the PDC. Thermogravimetry (TG) coupled with differential thermal analysis was used for understanding of the conversion/decomposition phenomena, and was subsequently used for the definition of an optimal heat‐treatment routine (Fig*.* 1). Melting of UHMW‐PE started in the temperature range 135°C–140°C (being in good accordance with both TMA results, see Section 3.3, and manufacturer datasheet), whereas the onset of the decomposition of UHMW‐PE was observed at 460°C. At 500°C, decomposition of UHMW‐PE was complete, leaving no solid residue. For the preceramic PSZ component, thermal conversion reactions involving the release of gaseous species were most pronounced in the temperature region between 300°C and 600°C. No significant mass change was observed beyond 800°C.

Based on these observations, a tailored pyrolysis schedule was developed, including an isothermal hold at 130°C to achieve full curing before melting of the UHWM‐PE fillers, a reduction in the heating rate starting at 300°C to allow for the release of gaseous species of both the preceramic polymer and the sacrificial fillers, and a final hold at a pyrolysis temperature of 800°C for 4 h to stabilize the material.

### 3.2 Screening of Parameter Variations

**Figure 2:**
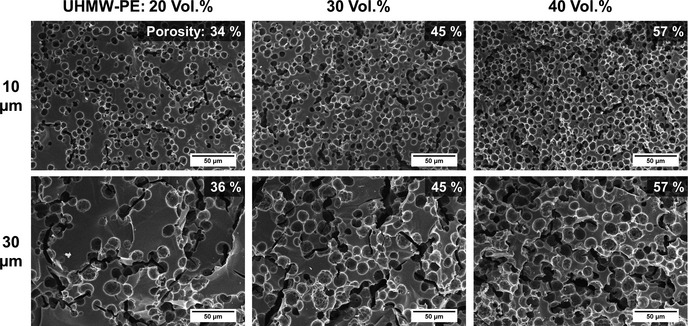
Fracture surfaces of PSZ/UHMW‐PE‐derived supports showing the sample morphology as a function of sacrificial filler particle size and volume fraction, as well as calculated average porosity values; samples cross‐linked at 90°C.

A variation in both the UHMW‐PE volume fraction and particle size in the initial mixtures resulted in materials with distinctly different physical properties and morphologies (Fig*.* 2). No residues of the UHMW‐PE component can be found in the generated pores after thermal treatment. In all materials, the presence of cracks can be observed in the fracture surfaces after pyrolytic conversion of samples cross‐linked at 90°C. Crack formation is particularly pronounced in samples with low UHMW‐PE contents, as a result of the extensive shrinkage of the PSZ during thermal conversion. Even though crack propagation appears to be hindered by the spherical pores, a further reduction in crack formation is desired for the anticipated applications.

The spherical pores are interconnected both by cracks and by pore windows, created by adjacent sacrificial filler particles before thermal decomposition, resulting in an open pore network.

The porosities of the materials, calculated from the sample dimensions, sample weight, and skeletal density (2.0 g/cm^3^, as determined by mercury intrusion porosimetry), are significantly higher than the volume fraction of the UHMW‐PE component in the initial mixture. Potential explanations for this behavior include the formation of additional porosity within the matrix during the pyrolytic conversion, the presence of gas bubbles due to incomplete wetting of the UHMW‐PE filler, or the presence of cracks.

**Figure 3:**
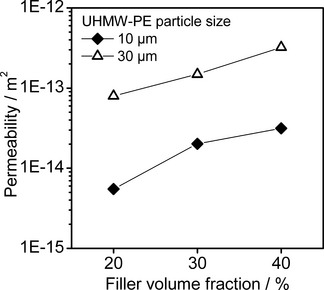
Air permeability of PSZ/UHMW‐PE‐derived supports as a function of sacrificial filler particle size and volume fraction; samples cross‐linked at 90°C (screening study).

For potential applications as membrane or catalysis supports, both permeability and strength characteristics are of major interest. With increasing UHMW‐PE content and size, a significant increase in air permeability through the material was observed (Fig*.* 3), with differences in permeability between sample compositions spanning two orders of magnitude. As the porosities of pyrolyzed specimens were well beyond 30% and hence above the reported percolation threshold of spherical pores,^46^ no phenomena associated with the transition from isolated pores to an interconnected pore network (e.g., abrupt increase in permeability) were observed in the parameter range investigated.

**Figure 4:**
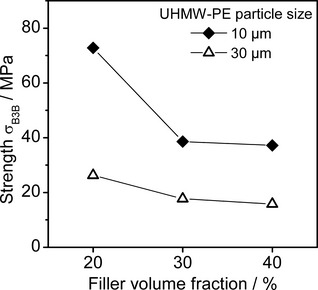
Biaxial flexural strength of PSZ/UHMW‐PE‐derived supports as a function of sacrificial filler particle size and volume fraction; samples cross‐linked at 90°C. Data points represent the average strength value (screening study).

A screening of the strength of samples showed an opposing trend compared to permeability characteristics, samples with smaller UHMW‐PE particles and lower porogen contents having a tendency toward higher strength values (Fig*.* 4). Even though samples with 10 and 30 μm‐sized UHMW‐PE had comparable total porosities, materials derived from smaller filler particles exhibited significantly higher strength values, most likely as a result of decrea‐sed defect size of both cracks and pores. However, the small number of samples investigated in these screening tests only yields limited results in terms of statistical significance. Thus, a more in‐depth characterization of promising sample compositions is presented in the following section.

### 3.3 Optimization of the Microstructure and In‐Depth Characterization of Performance

Based on the preliminary screening tests, PSZ with 30 vol% UHMW‐PE (10 μm) was chosen for further investigations due to the combination of its strength and permeability characteristics, appearing to be well‐suited for the intended application as a support structure.

**Figure 5:**
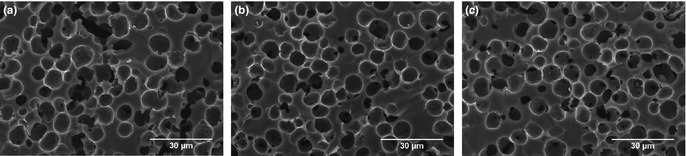
Comparison of the microstructure of pyrolyzed PSZ/UHMW‐PE‐derived supports cross‐linked at (a) 90°C, (b) 105°C, and (c) 120°C (30 vol% UHMW‐PE, 10 μm).

**Figure 6:**
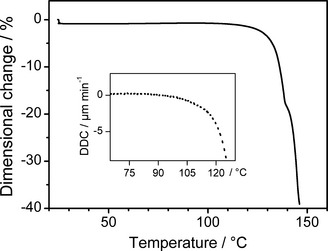
Thermomechanical analysis of a pressed UHMW‐PE pellet, showing the dimensional change in the material during heating. The insert, representing the derivative of dimensional change (DDC), shows the softening of the material, starting at around 100°C.

As a first step, the tendency of crack formation during pyrolytic conversion was addressed. By increasing the cross‐linking temperature of the PSZ/UHMW‐PE mixture, the number of cracks in the material was significantly reduced (Fig*.* 5). Two potential reasons for this behavior are postulated. First, a softening of the UHMW‐PE phase with an onset at around 100°C was observed by TMA investigations (Fig*.* 6), possibly leading to decreased stress build up during the cross‐linking step as a result of thermal expansion differences between the preceramic polymer and sacrificial filler phases. Second, a more thorough completion of the cross‐linking reaction of the polymer‐derived matrix phase—as a result of higher temperatures—leads to higher mechanical strengths in green state. However, this phenomenon also results in an increased brittleness of the material, thus potentially promoting the introduction of secondary cracks and surface defects during the machining in green state, and, subsequently, negatively affecting the overall performance.

**Figure 7:**
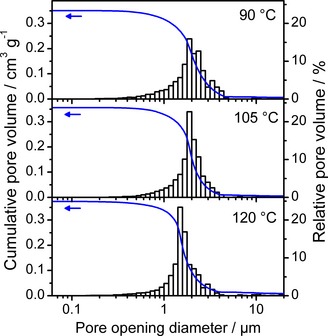
Pore opening size distribution as a function of cross‐linking temperature, determined by mercury intrusion porosimetry on pyrolyzed samples (30 vol% UHMW‐PE, 10 μm; data for 105°C partially reproduced from Ref. 42).

As the pore structure of the materials significantly affects the performance of the materials, mercury intrusion porosimetry was used for further clarification. While the density and total porosity of specimens was largely unaffected by the variations in cross‐linking temperature (Table 1), differences in the median pore diameter and shape of the pore size distribution were observed (Fig*.* 7). Both a decrease in median pore diameter and a narrowing of the pore size distribution after increasing the cross‐linking temperature were found.

**Table 1:** Density and Porosity Data of Pyrolyzed Materials (30 vol% UHMW‐PE, 10 μm) as a Function of Cross‐Linking Temperature, Determined by Mercury Intrusion Porosimetry

Cross‐linking temperature (°C)	90	105	120
Bulk density (g/cm^3^)	1.17	1.18	1.15
Skeletal density (g/cm^3^)	2.00	2.04	2.02
Porosity (%)	41.6	42.0^b^	43.2
Median pore diameter^a^ (μm)	2.0	1.9^b^	1.6

a By volume.

b Data previously published in Ref. 42.

The pore diameters determined by porosimetry correspond to the pore window size which is well below the actual size of the spherical pores in the range 7–8 μm. As the permeability is largely controlled by the pore windows, a correlation between pore window size and permeability can be observed, resulting in lower permeabilities at higher cross‐linking temperatures (Table 2). Both the wider distribution of pore opening diameters and the significantly higher permeability of samples cross‐linked at 90°C can be attributed to a higher number of cracks present in the material with these processing conditions.

**Table 2:** Strength and Permeability Characteristics of Pyrolyzed PSZ/UHMW‐PE‐Derived Ceramic Supports (30 vol% UHMW‐PE, 10 μm) as a Function of Cross‐Linking Temperature and Postpyrolysis Long‐Term Heat Treatment

Cross‐linking temperature/°C	Postpyrolysis heat treatment	Characteristic strength σ_0_ a/MPa	Weibull modulus *m* a	Permeability (×10^−15^)/m²
90	—	37.4 (33.8–41.5)	4.2 (3.0–5.8)	20.2 ± 6.9
105	—	65.7 (58.9–73.5)	4.1 (2.9–5.9)	9.5 ± 1.5^b^
120	—	54.4 (39.1–76.4)	1.5 (1.0–2.3)	6.3 ± 2.0
105	100 h, air	60.7 (50.5–73.2)	2.2 (1.6–3.1)	11.0 ± 3.5
105	100 h, N_2_	59.5 (50.3–70.6)	2.5 (1.8–3.5)	12.2 ± 3.1
105	100 h, N_2_/5% H_2_	55.0 (45.7–66.3)	2.3 (1.7–3.2)	8.3 ± 3.1

a For strength and modulus, ranges in brackets refer to the 90% confidence interval.

b Data previously published in Ref. 42.

The strength of specimens was evaluated by Weibull analysis of biaxial flexural strength data (Table 2). Samples cross‐linked at 105°C showed the maximum characteristic strength of 65.7 MPa. Even though cross‐linking at 90°C led to lower strength values as a result of cracks in the material, the Weibull modulus of the two sample batches was comparable. However, further increase in temperature to 120°C during cross‐linking resulted in a decreased characteristic strength and, even more pronounced, a lower Weibull modulus. This represents a higher variability in strength data and, correspondingly, a decrease in sample reproducibility. A possible reason for this behavior is the increased brittleness of the preceramic polymer material after cross‐linking at higher temperatures, potentially leading to the introduction of surface defects during machining in green state. Due to the small dimensions and high surface‐to‐volume ratio of the prepared samples, surface defects are expected to be the dominant origin of failure. As a result, the formation of surface defects in specimens during machining potentially counteracts any strength improvements achieved by the suppression of microcrack formation in the sample bulk through cross‐linking at higher temperatures, thus negatively affecting the overall mechanical properties.

Based on these results, a cross‐linking temperature of 105°C appears to yield optimal results for the materials combination used, leading to specimens with high mechanical strength, high reproducibility, as well as suitable permeability.

In light of the experimental results demonstrating the influence of the pore structure on the mechanical properties, an approach to tailor the evolution of microcracks—and, subsequently, the mechanical performance—through control of the pore structure seems obvious. While it is well‐known that, in addition to total porosity, both pore size and pore shape have a significant impact on the fracture of ceramic materials,^47^ in the present work, a correlation is nontrivial due to several factors discussed before, including cross‐linking‐induced intercomponent stresses, matrix shrinkage, and gas evolution during filler decomposition/polymer pyrolysis. All listed phenomena possibly affect the crack formation tendency, and hence would have to be taken into account for future approaches to tailoring the microcrack structure.

### 3.4 High‐Temperature Stability of Specimens in Various Atmospheres

**Figure 8:**
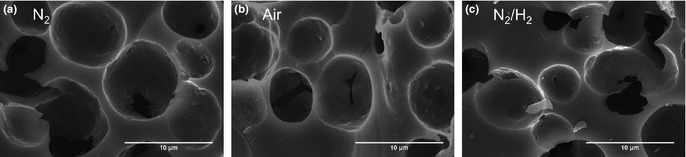
Comparison of the microstructure of PSZ/UHMW‐PE‐derived supports after heat treatment in (a) N_2_, (b) air, and (c) N_2_/H_2_ at 800°C for 100 h.

To test the performance of the material for potential applications at high temperatures, long‐term heat treatments at 800°C for 100 h in oxidizing (air), inert (N_2_), and reducing atmosphere (N_2_/H_2_) were conducted, comparing pyrolyzed sample batches of the most promising material/method combination (PSZ with 30 vol% UHMW‐PE, 10 μm‐sized, cross‐linked at 105°C). No significant changes in microstructural features were found after long‐term heat treatments (Fig*.* 8).

**Figure 9:**
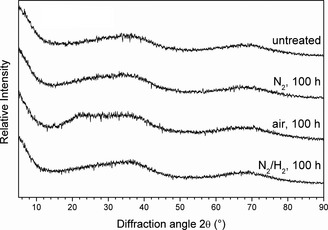
X‐ray diffraction patterns of PSZ/UHMW‐PE‐derived supports after pyrolytic conversion and after heat treatment in N_2_, air, and N_2_/H_2_ at 800°C for 100 h.

In all three cases, the samples exhibited an additional linear shrinkage of 1.5% during the long‐term heat‐treatment step (compared to a linear shrinkage of around 24% during the initial polymer‐to‐ceramic conversion step), suggesting further rearrangements within the polymer‐derived ceramic phase. Weight change during long‐term heat treatment was negligible (≤0.1%), with the exception of samples treated in air (+2.0%). X‐ray diffraction analysis showed no crystalline phases in the material before or after long‐term heat treatment (Fig*.* 9). While diffraction patterns after exposure to N_2_ or N_2_/H_2_ resembled the untreated material, heat treatment in air resulted in the evolution of an additional amorphous bump at a diffraction angle of around 23° which can be explained by the presence of amorphous silica as a result of oxidation processes.

While the characteristic strength and permeability of samples are largely unaffected by the heat treatment, a slight decrease in Weibull modulus appears to be present in comparison to the untreated pyrolyzed samples, even though the 90% confidence intervals slightly overlap (Table 2). No significant differences in characteristic strength, Weibull modulus, or permeability were found between samples heat‐treated in different atmospheres. These results suggest a high suitability of the generated structures for applications in various atmospheres at elevated temperatures, without the risk of major changes in material composition, pore structure, or performance during operation.

## 4 Conclusions

In this work, we have presented the preparation of novel porous ceramic support structures based on the tailoring of the pore structure in polysilazane‐derived silicon carbonitride ceramics by a sacrificial filler approach, employing spherical UHMW‐PE particles as porogens.

The straightforward preparation procedure involving mixing of components, casting at ambient pressure, and thermal cross‐linking was found to reproducibly yield dense, bubble‐ free specimens, without the need for time‐consuming consolidation techniques such as warm‐pressing. Therefore, it is a promising method for the generation of porous supports of not only planar geometry, but also for specimens with other, more complex shapes.

A subsequent pyrolytic conversion at relatively low temperatures (in comparison to conventional consolidation temperatures of non‐oxide ceramics) resulted in the generation of porous non‐oxide ceramic materials with interconnected, well‐defined porosity. A careful control of the temperature regime during cross‐linking was found to be essential in the suppression of crack formation in the sample bulk during the subsequent pyrolysis step, with an optimum cross‐linking temperature found to be 105°C.

A variation in the content and the size of sacrificial filler particles significantly affected the physical properties. This, in turn, allows for a tailorability of the pore structure and, ultimately, a control of the permeability and strength characteristics fitted to the intended application requirements.

In the pyrolyzed state, the generated porous materials exhibited long‐term stability at elevated temperatures in oxidizing, inert, and reducing atmospheres without significant decline in materials performance, thus being well suited for potential uses as membrane or catalysis supports in a wide range of typical application environments.
